# Possible association of decreased NKG2D expression levels and suppression of the activity of natural killer cells in patients with colorectal cancer

**DOI:** 10.3892/ijo.2011.1315

**Published:** 2011-12-22

**Authors:** YAJUAN SHEN, CHAO LU, WENJUN TIAN, LAICHENG WANG, BIN CUI, YULIAN JIAO, CHUNYAN MA, YING JU, LING ZHU, CHUNHONG SHAO, XINQI LIU, JIAN WANG, BINGCHANG ZHANG, ZHIMING LU

**Affiliations:** 1 Department of Clinical Laboratory, Shandong Provincial Hospital Affiliated to Shandong University, Jinan 250021, P.R. China; 2 Department of Central Laboratory, Shandong Provincial Hospital Affiliated to Shandong University, Jinan 250021, P.R. China; 3 Department of Respiratory Medicine, Shandong Provincial Hospital Affiliated to Shandong University, Jinan 250021, P.R. China

**Keywords:** NKG2, natural killer cells, colorectal cancer, suppression

## Abstract

Natural-killer group 2 (NKG2), a natural killer (NK) cell receptor, plays a critical role in regulating NK cytotoxicity. In this study, we investigated the expression levels of natural killer group 2 member A (NKG2A) and natural killer group 2 member D (NKG2D) in NK cells as well as the regulatory function of NKG2D in patients with colorectal cancer (CRC). Sixty-two CRC patients and 32 healthy controls were enrolled in this study. The expression levels of NKG2A and NKG2D mRNA in peripheral blood mononuclear cells (PBMCs) were investigated using real-time PCR. Flow cytometry was performed to assay the levels of NKG2A and NKG2D proteins in NK cells. The levels of NKG2D mRNA in PBMCs in the patients were significantly lower than those in the controls [mean ± SD, 1.11±0.60 (CRC patients) vs. 1.65±0.71 (healthy controls); p<0.01], whereas the 2 groups showed no apparent difference in the levels of NKG2A mRNA (p>0.05). In addition, the patients showed significantly lower NKG2D levels in NK cells than the controls did (71.23%±8.31% [CRC patients] vs. 79.39%±5.58% [healthy controls]; p<0.01). However, we observed no distinct difference in the NKG2A expression levels in NK cells between the 2 groups (p>0.05). Notably, blockage of NKG2D signaling with anti-NKG2D antibodies ex vivo resulted in decreased cytotoxicity and CD107a degranulation. Our data revealed that the decrease in NKG2D expression levels may have been associated with suppression of NK cell activity in CRC patients.

## Introduction

Recent statistics show that colorectal cancer (CRC) is the third most commonly diagnosed cancer in men and the second in women worldwide, with millions of new cancer cases being reported annually. CRC is also the third most fatal cancer, causing ~600,000 deaths annually (1–4). However, the precise mechanisms of immune suppression in CRC are not yet completely understood.

Natural killer (NK) cells play a critical role in innate immunity against viral infections and tumors. The functions of NK cells are regulated by the integration of signals from inhibitory and activating receptors (5). When activating signals are predominant, NK cells are activated, and they show cytotoxic activity and secrete cytokines. However, when inhibitory signals are predominant, NK cells are not activated and do not show antitumor immune responses.

Natural-killer group 2 member A (NKG2A) and natural-killer group 2 member D (NKG2D), both of which belong to the C-type lectin superfamily, are a pair of vital inhibitory and activating receptors, respectively, in NK cells. Previous studies have suggested that these receptors may be expressed in other immune cells such as T and NKT cells (6–9). The interaction between NKG2A/NKG2D and its ligands has been linked to a wide variety of physiologic and pathologic functions (10–26). Much effort has been devoted to understanding the roles of these proteins in the regulation of activities of immune cells and importance in immune responses. However, little is known about the role of NKG2A/NKG2D in colorectal cancer. In this study, we examined the expression of NKG2A/NKG2D in NK cells and determined the role of the NKG2 pathway in the regulation of NK cytotoxicity in patients with CRC.

We examined the expression of NKG2A and NKG2D in peripheral blood mononuclear cells (PBMCs) and NK cells from patients with CRC by using real-time PCR and flow cytometry. Furthermore, we assessed the functions of NK cells by using cytotoxicity assay and CD107a degranulation assay. We found that the NKG2D expression levels were significantly lower in the CRC patients than in the healthy controls, whereas NKG2A expression levels in the CRC patients were similar to those in the healthy controls. We also found that NK cell activity declined when NKG2D signaling was blocked with anti-NKG2D antibodies. Therefore, we concluded that the decrease in NKG2D expression may be connected to NK cell suppression in patients with CRC. Thus, our study provides a basis for the mechanism underlying the escape of tumor cells from immune surveillance *in vitro*.

## Materials and methods

### Patients and controls

Sixty-two patients (34 men and 28 women) with primary CRC were recruited from the gastrointestinal surgery ward of Shandong Provincial Hospital, China. These patients were diagnosed with CRC on the basis of colorectal cancer diagnosis standard (2010) issued by Ministry of Health, China. The patients had no history of other diseases such as heart disease, diabetes, kidney disease, or autoimmune disease. Thirty-two healthy subjects (18 men and 14 women) from the physical examination centre of Shandong Provincial Hospital. Clinical characteristics of the enrolled subjects are summarized in Table I. All individuals included in this study were unrelated and randomly selected. The study was approved by Shandong University Ethics Committee, and informed consent was acquired from each individual.

### Cells and cell culture

PBMCs were isolated from venous blood obtained from CRC patients before surgery and from healthy subjects by using Ficoll-Hypaque density gradient centrifugation (Tianjin Haoyang Bio Co. Ltd., China).

NK cells were isolated from PBMCs by positive selection by using magnetic cell separation (Miltenyi Biotec, Germany) according to the manufacturer’s instructions. Flow cytometry revealed the purity of CD3^-^CD56^+^ NK cells to be >95%. For functional assays, the obtained NK cells were cultured in RPMI-1640 medium (Invitrogen, China) with 10% heat-inactivated fetal calf serum (FCS) (Invitrogen) and 100 U/ml recombinant human interleukin 2 (rIL-2) (Sangon Bio Co. Ltd., China).

Human colon carcinoma cell line HT29 was kindly provided by Professor Zhang Jian, School of Pharmaceutical Science, Shandong University. HT29 cells were cultured in RPMI-1640 supplemented with 10% FCS and were used as target cells. The medium was regularly changed, and the cells were always washed twice before use.

Neutralizing antibodies against NKG2D (R&D Systems, USA) were used in antibody-blocking experiments. Purified NK cells were pre-incubated with 10 μg/ml anti-NKG2D antibodies for 30 min before they were cultured with the target cells. Then, the cytotoxicity and CD107a degranulation assays were performed.

### Real-time PCR

Total cellular RNA was extracted from PBMCs by using TRIzol reagent (Invitron, USA) according to the manufacturer’s instructions. Concentration and quality of the extracted total RNA were determined by measuring its light absorbance at 260 nm (A260) and the ratio of (A260/A280). A 1 μg of total RNA was reverse transcribed in a 20-μl reaction mixture containing 2 μl of Maxima enzyme mix (Fermentas, Canada) and 4 μl of 5X PCR mix (Fermentas). The procedure for reverse transcription was performed as follows: 10 min at 25°C, 30 min at 55°C, and then 5 min at 85°C. All these procedures were performed using the GeneAmp PCR system 2720 (Applied Biosystems, USA). The obtained cDNA was diluted 1:10 before PCR analysis.

The primers used for CD94, NKG2A, NKG2D, and β-actin are shown in Table II. All the primers were synthesized and validated by Sangon Bio Co. Ltd. Reactions were performed in a total volume of 20 μl, which included 5 μl of cDNA sample, 5 μl of 0.8 μM primer pair, and 10 μl of 2X PCR mix (Takara, Japan). PCR was performed as follows: 5 min at 95°C and 45 cycles of 15 sec at 95°C, 30 sec at 60°C, and 15 sec at 72°C. Incubation and on-line detection of the PCR products were carried out using optical 96-well plates and the LightCycler 480 sequence detection system (Roche, Germany). Finally, the PCR products were subjected to electrophoresis to determine whether the required products were formed.

### Flow cytometry

PBMCs were washed with phosphate-buffered saline (PBS) containing 2% FCS and stained with anti-CD3-PC5 (Beckman Coulter, USA), anti-CD56-FITC (Beckman Coulter), anti-NKG2A-PE (Becton-Dickinson, USA), and anti-NKG2D-PE (Becton-Dickinson) antibodies. At least 10,000 cells were analyzed using a 3-color EPICS XL flow cytometer (Beckman Coulter).

For CD107a degranulation assays, NK cells and HT29 cells were co-cultured for 4 h at 37°C. Then, they were washed with PBS and stained with anti-CD3-PC5, anti-CD56-FITC, and anti-CD107a-PE antibodies (Becton-Dickinson). Expression of CD107a on CD3-CD56^+^ NK cells was analyzed by using the EPICS XL flow cytometer.

### Cytotoxicity assays

HT29 cells were used as target cells, and the purified NK cells were used as effector cells in the ^51^Cr release assay. The HT29 cells were labeled by incubating them with the ^51^Cr isotope for 1 h at 37°C. The labeled targets were then co-incubated with NK cells at different ratios for 4 h. The supernatant was harvested and analyzed using a γ-counter (Packard Cobra II 5002, USA). The percentage of ^51^Cr released (counts per min, c.p.m.) was calculated as follows: [(experimental release - spontaneous release)/(maximum release - spontaneous release)] ×100%. Spontaneous release was <15% of the maximum release. All experiments were performed in triplicates.

### Statistical analysis

A t-test was used for comparing the findings of the patient group and the control group. A one-way Anova was used for comparing the data of three or four groups. Probability values were considered significant at p<0.05. Statistical analyses were performed by using the GraphPad Prism 4 (GraphPad Software, USA).

## Results

### Expression levels of NKG2A mRNA in the CRC patients were similar to those in the healthy controls, whereas those of NKG2D mRNA in the PBMCs of the CRC patients were lower than those in the healthy controls

**Figure 1:**
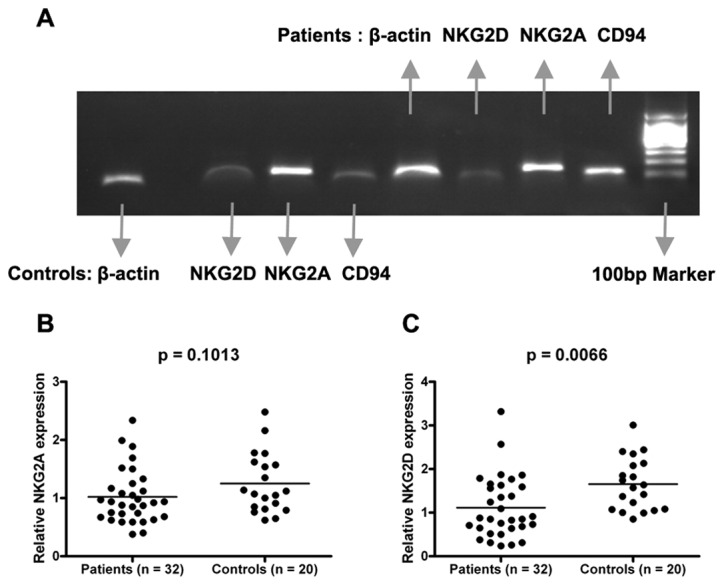
Expression levels of NKG2A were similar in the CRC patients and healthy controls, and those of NKG2D in the PBMCs were lower in the CRC patients than in the healthy controls, as determined by real-time PCR. (A) Electrophoresis stripe of CD94, NKG2A, NKG2D and β-actin by real-time RT-PCR. (B) Relative expression levels of NKG2A and (C) NKG2Din PBMCs from CRC patients and healthy controls determined by using real-time PCR analysis, were compared. Each dot represents a subject.

To evaluate the expression levels of NKG2A and NKG2D mRNA in PBMCs, we performed real-time PCR analysis for 32 CRC patients and 20 healthy subjects. There was no difference in NKG2A expression levels in PBMCs in the CRC patients and in the healthy controls [mean ± SD, 1.02±0.47 (CRC patients) vs. 1.25±0.52 (healthy controls)]; p>0.05] (Fig. 1B). Instead, there was a significant decline in NKG2D expression in the CRC group [1.11±0.60 (CRC patients) vs. 1.65±0.71 (healthy controls); p<0.01] (Fig. 1C). Taken together, these results indicated that the expression of inhibitory receptor NKG2A mRNA did not change, whereas that of the activating receptor NKG2D mRNA decreased in CRC patients.

### Expression levels of NKG2A protein were similar in the CRC patients and healthy controls, whereas those of NKG2D protein in the PBMCs were lower in the CRC patients than in the healthy controls

**Figure 2:**
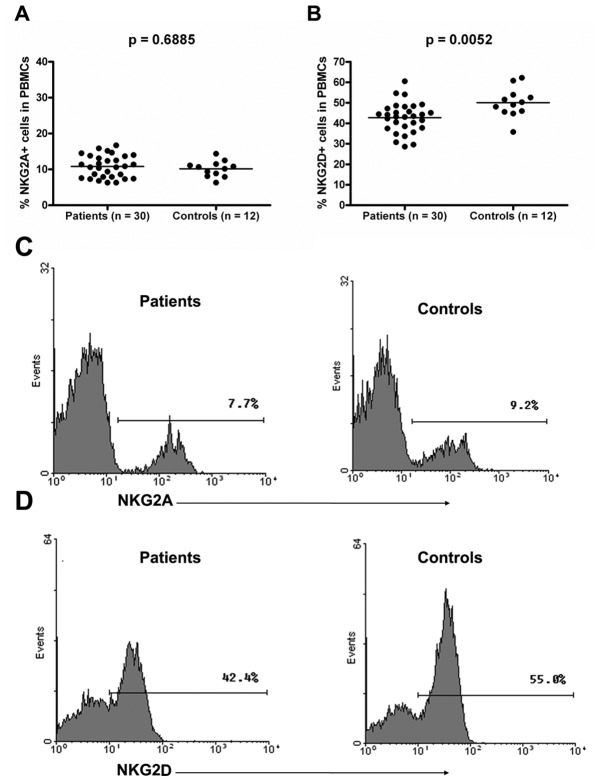
NKG2A protein levels were similar in the CRC patients and healthy controls, and NKG2D protein levels in the PBMCs were lower in the CRC patients than in the healthy controls. (A) Levels of NKG2A and (B) NKG2D expression in PBMCs from patients and healthy controls were determined by flow cytometric analysis. Each dot represents a subject. (C) Representative histograms of NKG2A and (D) NKG2D levels in PBMCs from CRC patients and healthy controls.

Flow cytometry studies in 30 CRC patients and 12 healthy subjects showed that NKG2A protein levels were similar in both the groups [10.83%±3.11% (CRC patients) vs. 10.15%±2.20% (healthy group); p>0.05] (Fig. 2A and C). However, the NKG2D protein levels were significantly lower in the CRC patients than in the healthy controls [42.70%±7.35% (CRC group) vs. 50.06%±7.13% (healthy groups); p<0.01] (Fig. 2B and D). These results indicated that the expression levels of NKG2A protein were similar in both the groups, whereas those of NKG2D protein were lower in the CRC patients than in the healthy controls.

### Although NKG2A expression levels were similar in the CRC patients and healthy controls, NKG2D expression levels in NK cells were lower in the CRC patients than in the healthy controls

**Figure 3:**
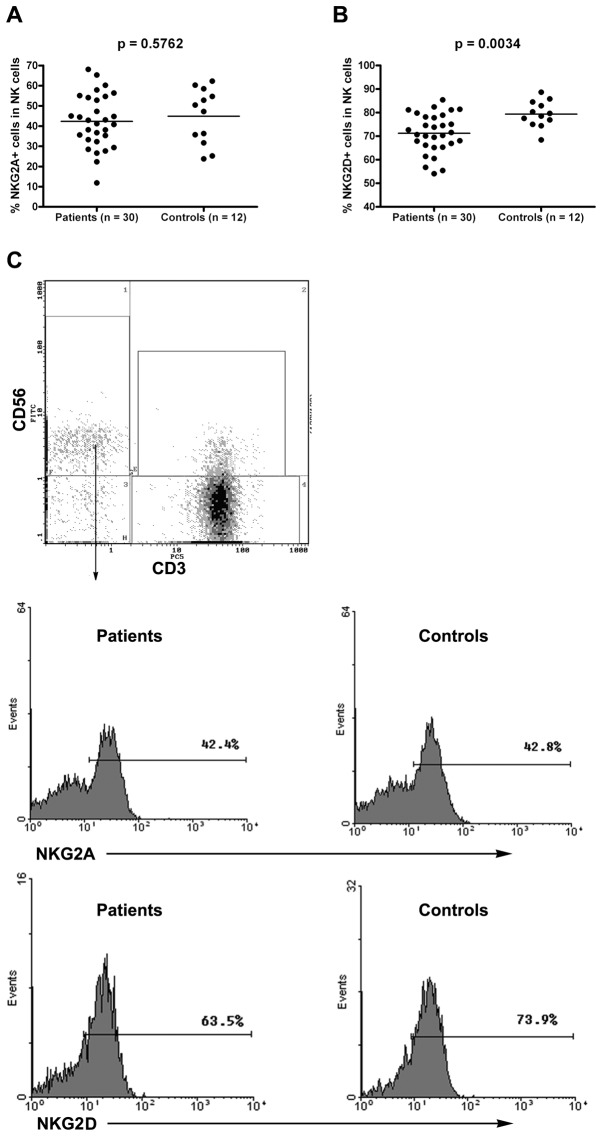
NKG2A expression levels were similar in the CRC patients and healthy controls, and NKG2D expression levels in the NK cells were lower in the CRC patients than in the healthy controls. (A) Levels of NKG2A and (B) NKG2D expression in CD3^−^CD56^+^ NK cells from patients and healthy controls determined by using flow cytometry, were compared. (C) Representative histogram plots of NKG2A and NKG2D expression in CD3^−^CD56^+^ NK cells from CRC patients and healthy controls.

NKG2A expression levels in NK cells were similar in both the groups [42.34%±13.20% (CRC group) vs. 44.91%±13.77% (healthy group); p>0.05] (Fig. 3A and C). However, the number of NKG2D^+^ NK cells in the CRC patients was significantly lower than that in the healthy controls [71.23%±8.31% (CRC group) vs. 79.39%±5.58% (healthy group); p<0.01] (Fig. 3B and C). These results indicated that, although the expression of NKG2A in NK cells was similar in both groups, those of NKG2D in NK cells were lower in the CRC than in the healthy controls.

### Blocking NKG2D expression reduces NK cytotoxicity and CD107a degranulation

**Figure 4:**
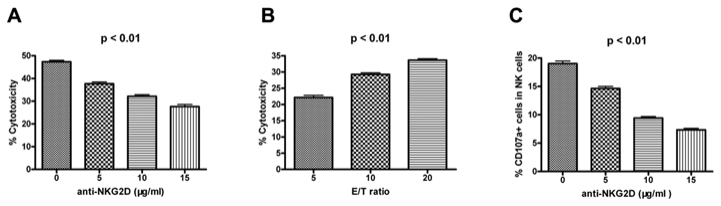
Blocking NKG2D expression reduced NK cytotoxicity and CD107a degranulation. (A) The effect of the indicated doses of anti-NKG2D antibodies on NK cytotoxicity was measured on the basis of the percentage of HT29 cells killed at an effector to target ratio (E/T) of 20:1. (B) NK cytotoxicity was determined by the percentage of HT29 cells killed at the indicated E/T ratio after pre-incubation of the cells with anti-NKG2D antibodies at a concentration of 10 μg/ml. (C) The effect of the indicated doses of anti-NKG2D antibodies on the NK degranulation was determined on the basis of the percentage of CD107a^+^ CD3^−^CD56^+^ NK cells after incubation with HT29 cells at an E/T ratio of 20:1. Statistical significance (p<0.05) for the specific lysis among different groups was determined using a one-way Anova. All results shown are representative of the 4 triplicate samples.

In order to investigate the role of NKG2D in NK cells, we used anti-NKG2D antibodies to block the NKG2D pathway in an NK cell-mediated cytotoxicity assay. NK cytotoxicity (Fig. 4A and B) and CD107a degranulation (Fig. 4C) decreased with an increase in the concentration of anti-NKG2D antibody. These results suggested that NKG2D could activate NK cell functions. Therefore, a decrease in NKG2D expression levels would result in a decrease in the level of NK cell activity.

### Expression of NKG2A and NKG2D in NK cells, NKT cells, and T cells

**Figure 5:**
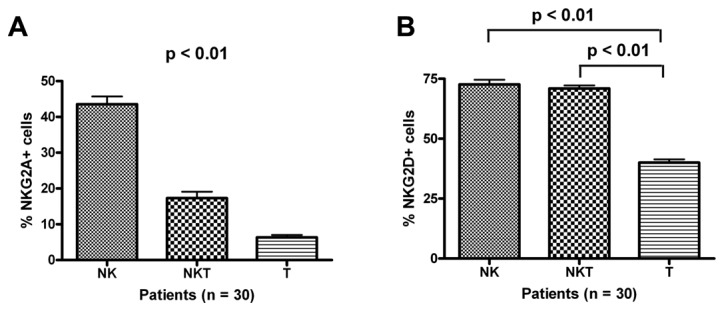
NKG2A and NKG2D expression in NK cells, NKT cells, and T cells. (A) Levels of NKG2A and (B) NKG2D expression in CD3^−^CD56^+^ NK cells, CD3^+^CD56^+^ NKT cells, and CD3^+^CD56^−^ T cells from patients (n=30), as determined by flow cytometry, were compared.

We also found that NKG2A and NKG2D were expressed on the membranes of CD3^+^CD56^−^ T and CD3^+^CD56^+^ NKT cells. However, NKG2A expression levels decreased successively in NK cells, NKT cells, and T cells (Fig. 5A; p<0.01). The NKG2D expression levels in T cells were lower than the corresponding levels in NK cells and NKT cells (Fig. 5B; p<0.01).

## Discussion

In this study, we first detected the expression of NKG2A and NKG2D by using real-time PCR. We found that NKG2A expression levels were similar in both groups, whereas NKG2D expression levels were significantly lower in the CRC patients than in the healthy controls. These findings suggest an imbalance in NKG2A/NKG2D expression at the transcription level in CRC patients.

Next, we detected NKG2A and NKG2D expression in PBMCs by flow cytometry and found that NKG2A expression levels in PBMCs and NK cells in the CRC patients were similar to the corresponding levels in the healthy controls. However, NKG2D expression levels in not only PBMCs but also NK cells were significantly lower in CRC patients than in the healthy controls. A similar kind of decrease in NKG2D expression levels was observed in patients with hepatic carcinoma (27). Therefore, these findings suggest that NKG2D expression may be altered at the translation level in CRC patients. Taken together, the balance of inhibitory NKG2A and activating NKG2D receptors shifted in CRC patients. Once the NKG2 receptors recognize and combine with their corresponding ligands on tumor cells, inhibitory signals are generated predominantly. NKG2D ligands MICA, MICB, and ULBPs and NKG2A ligand HLA-E may be expressed in CRC patients (13,15,23,27). HLA-E expression on the surface of tumor cells may allow the tumor cells to escape the immune surveillance by T and NK cells (13,15,22).

How does this shift affect the functions of NK cells in CRC patients? We assessed NK cell-mediated cytotoxicity and CD107a degranulation and found NK cytotoxicity and CD107a degranulation dropped to a lower degree when NKG2D expression was blocked in CRC patients. These results are in accordance with the results of previous studies on other tumors (18,28,29). Therefore, we concluded that NKG2D plays an important role in activating NK cells, and the decrease in NKG2D expression level may result in decline of the activity of NK cells in CRC patients. Thus, NK cell-mediated antitumor immune responses may be inhibited via the NKG2 pathway in CRC patients. Recent studies have tried to illustrate the role of this pathway in spontaneous malignancy in NKG2D-deficent animal models (17).

Previous studies have stated that tumor cells may escape immune surveillance through a variety of mechanisms, despite innate and adaptive immunity in humans. Our study shows that the imbalance of NKG2A and NKG2D expression may mediate the suppression of NK cell activity in CRC patients, thereby contributing to the escape of tumor cells from NK-mediated lysis. It has been reported that some cytokines, for instance, IL-2, IL-12, IL-15, and IFN-α, may change NKG2 expression levels and enhance NK cell-mediated cytotoxicity (27,28,30). These studies provide a promising prospect for immunotherapy for carcinomas.

In conclusion, our results showed that decrease of NKG2D expression level in CRC patients may be associated with suppression of NK cell activity. We inferred that tumor cells may escape NK cell surveillance via the NKG2 pathway in CRC patients; however, the underlying mechanism needs to be investigated further in detail. The current study is a starting point in our continuous efforts to understand the NKG2 immune escape pathway and the pathogenesis of CRC. Our results may provide important insights for the development of anticancer strategies.

## Figures and Tables

**Table I:** Clinical characteristics of enrolled subjects.

Group	CRC	Healthy controls
Case	62	32
Gender (male)	34 (54.8%)	18 (56.3%)
Age (years)^a^	54±11.2	50±8.5

a Median ± SD.

**Table II:** Primer pairs used in real-time PCR analysis.

Primer	Sequence
CD94	
Forward	5′-TTG ATG GCT ACG TTG GGA ATT-3′
Reverse	5′-TTG GCA AGA ACA GCA GTC AGA-3′
NKG2A	
Forward	5′-TTG CTG GCC TGT ACT TCG A-3′
Reverse	5′-CCA AAC CAT TCA TTG TCA CCC-3′
NKG2D	
Forward	5′-TTC AAC ACG ATG GCA AAA GC-3′
Reverse	5′-CTA CAG CGA TGA AGC AGC AGA-3′
β-actin	
Forward	5′-ACA GAG CCT CGC CTT TGC C-3′
Reverse	5′-ACA TGC CGG AGC CGT TGT C-3′
